# Complete Genome Sequence of a Carbapenem-Resistant Extraintestinal Pathogenic *Escherichia coli* Strain Belonging to the Sequence Type 131 *H*30R Subclade

**DOI:** 10.1128/genomeA.00272-15

**Published:** 2015-04-09

**Authors:** Timothy J. Johnson, Melissa Hargreaves, Kristin Shaw, Paula Snippes, Ruth Lynfield, Maliha Aziz, Lance B. Price

**Affiliations:** a Department of Veterinary and Biomedical Sciences, University of Minnesota, St. Paul, Minnesota, USA; b Minnesota Department of Health, St. Paul, Minnesota, USA; c Division of Pathogen Genomics, the Translational Genomics Research Institute, Flagstaff, Arizona, USA; d Department of Occupational and Environmental Health, George Washington University, Washington, DC, USA

## Abstract

Here, we report the completed genome sequence of a carbapenem-resistant extraintestinal pathogenic *Escherichia coli* sequence type 131 (ST131) isolate, MNCRE44. The isolate was obtained in 2012 in Minnesota, USA, from a sputum sample from a hospitalized patient with multiple comorbidities, and it belongs to the *H*30R sublineage.

## GENOME ANNOUNCEMENT

Extraintestinal pathogenic *Escherichia coli*, or ExPEC, poses a significant burden on health care systems worldwide (1). Recently, ExPEC sequence type 131 (ST131) has emerged worldwide as the predominant ExPEC subtype isolated from human clinical cases (2). Within ST131, a subclade known as *H*30R is particularly concerning because of its reduced susceptibility to fluoroquinolones, and some of these isolates (*H*30Rx) also display reduced susceptibility toward third-generation cephalosporins (3). Recent reports of ExPEC ST131 isolates that are also resistant to carbapenems are rare, yet concerning (4–8).

ExPEC strain MNCRE44 was isolated in 2012 from the sputum sample from a patient with multiple comorbidities in Minnesota, and it was initially characterized as PCR positive for the *Klebsiella pneumoniae* carbapenemase (KPC)-encoding gene *bla*_KPC_ and displayed reduced susceptibilities to cefotaxime, ceftazidime, ciprofloxacin, ertapenem, and gentamicin. MNCRE44 was sequenced using a combination of PacBio (100× coverage, P5-C3 chemistry) and Illumina MiSeq (125× coverage, TruSeq chemistry) sequencing. The PacBio sequences were assembled using HGAP version 3 in the PacBio SMRT Portal, which resulted in closed circular plasmids and a circular chromosome. The PacBio sequence reads were manually error corrected using a MiSeq assembly from CLC Genomics Workbench version 7. The genome sequence was annotated using the NCBI Prokaryotic Genome Annotation Pipeline, followed by manual curation of the plasmid sequences. Antimicrobial resistance genes were identified using the CARD database (9). Plasmid replicons were identified using PlasmidFinder (10).

The complete genome of MNCRE44 contains a 5,010,884-bp chromosome, a G+C content of 50.8%, 5,407 coding sequences, 86 tRNAs, and 22 rRNA features. Six plasmids are present in MNCRE44 (pMNCRE44_1 to pMNCRE44_6) of sizes 1.5, 4.0, 5.1, 30, 116, and 122 kb, respectively. In addition to its chromosomally encoded fluoroquinolone resistance phenotype, MNCRE44 also possesses a 116-kb hybrid IncX3/IncFIA(HI1) plasmid (pMNCRE44_5) containing *sul2*, *aph(3′)-Ib*, *aph(6)-Id*, *bla*_TEM-1_, *bla*_OXA-9_, *aadA1*, *aacA4*, two copies of *bla*_KPC-3_, and *bla*_SHV-12_. Additionally, MNCRE44 possesses an IncFII(pRSB107)/FIA/FIB(AP001918) plasmid (pMNCRE44_6) containing a copy of *bla*_TEM-1_. MNCRE44 contains an IncX4 plasmid (pMNCRE44_4) that does not carry genes encoding resistance-associated elements. A phylogenetic comparison of the single nucleotide polymorphisms in the core genome of MNCRE44 compared to those in a known collection of ST131 isolates placed it in the *H*30R sublineage.

MNCRE44 is the first complete genome sequence for an *H*30R ST131 isolate that includes all plasmids. This is also the first completed sequence of a carbapenem-resistant ST131 ExPEC isolate. The genome sequence provides the genetic context related to the emergence of carbapenem resistance in ST131 ExPEC isolates.

### Nucleotide sequence accession numbers.

The complete sequences of the chromosome of *E. coli* MNCRE44 and its six plasmids, pMNCRE44_1 through pMNCRE44_6, have been deposited in GenBank (accession numbers CP010876, CP010877, CP010878, CP010879, CP010880, CP010881, and CP010882, respectively).
